# Supplementary material for: Dissemination and implementation of an evidence-based voluntary medical male circumcision program: The Spear & Shield program
Source: PLOS Glob Public Health. 2024 Jan 31;4(1):e0002065. doi: 10.1371/journal.pgph.0002065 (PMC10830021; doi:10.1371/journal.pgph.0002065)
Supplement: Attachment — Submitted filename: SS2 Main Paper RR.docx [file pgph.0002065.s003.docx]

Thank you for the opportunity to revise this manuscript. Below, we provide a detailed response to the reviewers along with revisions to the manuscript, and now have attached a de-identified version of the data. We hope these revisions are acceptable for publication in Plos One.

Reviewers' comments:

Reviewer's Responses to Questions

**Comments to the Author**

1. Does this manuscript meet PLOS Global Public Health’s [publication criteria](https://nam10.safelinks.protection.outlook.com/?url=https%3A%2F%2Fjournals.plos.org%2Fglobalpublichealth%2Fs%2Fcriteria-for-publication&data=05%7C01%7Cvjr5%40miami.edu%7Ccf5a94cc26fd4e54814508db5896e196%7C2a144b72f23942d48c0e6f0f17c48e33%7C0%7C0%7C638201177272720392%7CUnknown%7CTWFpbGZsb3d8eyJWIjoiMC4wLjAwMDAiLCJQIjoiV2luMzIiLCJBTiI6Ik1haWwiLCJXVCI6Mn0%3D%7C3000%7C%7C%7C&sdata=xoFTb1rn%2F%2FWa446ObtKiFkx0IFmFGaSqrC2sSefeNDY%3D&reserved=0)? Is the manuscript technically sound, and do the data support the conclusions? The manuscript must describe methodologically and ethically rigorous research with conclusions that are appropriately drawn based on the data presented.

Reviewer #1: Partly

Reviewer #2: Yes

2. Has the statistical analysis been performed appropriately and rigorously?

Reviewer #1: No

Reviewer #2: I don't know

3. Have the authors made all data underlying the findings in their manuscript fully available (please refer to the Data Availability Statement at the start of the manuscript PDF file)?

The [PLOS Data policy](https://nam10.safelinks.protection.outlook.com/?url=https%3A%2F%2Fjournals.plos.org%2Fglobalpublichealth%2Fs%2Fdata-availability&data=05%7C01%7Cvjr5%40miami.edu%7Ccf5a94cc26fd4e54814508db5896e196%7C2a144b72f23942d48c0e6f0f17c48e33%7C0%7C0%7C638201177272720392%7CUnknown%7CTWFpbGZsb3d8eyJWIjoiMC4wLjAwMDAiLCJQIjoiV2luMzIiLCJBTiI6Ik1haWwiLCJXVCI6Mn0%3D%7C3000%7C%7C%7C&sdata=3eb3KICIip35g%2B6NAd9aQsLqVvyZU1IGgy5WYGvPPNo%3D&reserved=0) requires authors to make all data underlying the findings described in their manuscript fully available without restriction, with rare exception. The data should be provided as part of the manuscript or its supporting information, or deposited to a public repository. For example, in addition to summary statistics, the data points behind means, medians and variance measures should be available. If there are restrictions on publicly sharing data—e.g. participant privacy or use of data from a third party—those must be specified.

Reviewer #1: Yes

Reviewer #2: No

4. Is the manuscript presented in an intelligible fashion and written in standard English?

PLOS Global Public Health does not copyedit accepted manuscripts, so the language in submitted articles must be clear, correct, and unambiguous. Any typographical or grammatical errors should be corrected at revision, so please note any specific errors here.

Reviewer #1: Yes

Reviewer #2: Yes

5. Review Comments to the Author

Please use the space provided to explain your answers to the questions above. You may also include additional comments for the author, including concerns about dual publication, research ethics, or publication ethics. (Please upload your review as an attachment if it exceeds 20,000 characters)

Reviewer #1: This is a very interesting manuscript describing the scale up of VMMC in 4 provinces of Zambia using the S&S and RE-AIM framework. In each province, clinic were divided into immediate (12), delayed (12) and observation groups (6) regarding VMMC (observation) and S&S (immediate and delayed). Result shows an increase of VMMC in the immediate clinics.

General: Clarity and organization of the paper can be improved to easy understanding. Write in full first time an acronym is used. review typos and totals.

**Response:** We have reviewed the entire paper again, corrected typos, reviewed totals, and spelled all acronyms in full the first time used.

Specific:
Methods


Ethics
1. Please considered a reference to the last version of the "Declaration of Helsinki 2013, Fortaleza, Brazil"

**Response:** We now cite the 2013 Declaration of Helsinki.

2. Please specify "written or oral consent"?

**Response:** We clarified written informed consent was obtained from all participants.

Procedures
1."conditions" and "Training" on 2nd and 3rd paragraphs respectively seem dislocated.

**Response:** We moved these sections around to improve readability.

2. "Trainees were GRZ lay counselors" please review sentence.

**Response:** We revised this sentence to say, “Trainees were lay counselors ­≥ 18 years old engaged in HIV prevention services in the Government of Republic of Zambia.”

Results
1. Says "N= 310", Actual sum =385
2. Table 1 : Please reorganize: "Employment at facilite (years)" - Less than 1 year, [2-3] etc,
3. Table 1: Be clear on what is presented: Age in years (mean, Median, SD, IQR)
4. Table 1: Be clear on what is presented: Monthly income in USD/ZK (mean, Median, SD, IQR)
5. Tabel 1: Include all acronyms in full in the figure legend caption
6. Ranges provided (average) > This is not clear - Mean or medium?

**Response:** We have revised Table 1 accordingly, confirmed numbers, and specified that Means and Standard Deviations are provided.

7. Figures need legends captions

**Response:** Captions and legends, now with greater detail, are presented at the end of the manuscript.

Discussion
1. Focus on results of this study not only the potential results.

**Response:** We removed references to the “potential” effects of the translating S&S in the Discussion, and instead focus on the results from the study.

Reviewer #2: Thank you for this opportunity to review this interesting article titled" Dissemination and implementation of an evidence-based voluntary medical male circumcision program: The Spear & Shield Program". The manuscript is technically sound and reaches a conclusion that is consistent with previous research. The study adds significant value to the global efforts aiming to contain HIV infections. Nonetheless, data supporting findings are required to be attached or deposited to a public repository.

**Response:** We have now attached an de-identified version of the data to the submission.

6. PLOS authors have the option to publish the peer review history of their article ([what does this mean?](https://nam10.safelinks.protection.outlook.com/?url=https%3A%2F%2Fjournals.plos.org%2Fglobalpublichealth%2Fs%2Feditorial-and-peer-review-process%23loc-peer-review-history&data=05%7C01%7Cvjr5%40miami.edu%7Ccf5a94cc26fd4e54814508db5896e196%7C2a144b72f23942d48c0e6f0f17c48e33%7C0%7C0%7C638201177272720392%7CUnknown%7CTWFpbGZsb3d8eyJWIjoiMC4wLjAwMDAiLCJQIjoiV2luMzIiLCJBTiI6Ik1haWwiLCJXVCI6Mn0%3D%7C3000%7C%7C%7C&sdata=jswYrqEnXF2srkwGsVioRSxGVylGz6Ig3sy6xUsAby0%3D&reserved=0)). If published, this will include your full peer review and any attached files.


**Do you want your identity to be public for this peer review?** If you choose “no”, your identity will remain anonymous but your review may still be made public.


For information about this choice, including consent withdrawal, please see our [Privacy Policy](https://nam10.safelinks.protection.outlook.com/?url=https%3A%2F%2Fwww.plos.org%2Fprivacy-policy&data=05%7C01%7Cvjr5%40miami.edu%7Ccf5a94cc26fd4e54814508db5896e196%7C2a144b72f23942d48c0e6f0f17c48e33%7C0%7C0%7C638201177272720392%7CUnknown%7CTWFpbGZsb3d8eyJWIjoiMC4wLjAwMDAiLCJQIjoiV2luMzIiLCJBTiI6Ik1haWwiLCJXVCI6Mn0%3D%7C3000%7C%7C%7C&sdata=mdL559v1ABXyU%2FGU2IqFctRs5weYWzxywszaHXvP%2BrU%3D&reserved=0).

Reviewer #1: No

Reviewer #2: No
