# Supplementary material for: Dissemination and implementation of an evidence-based voluntary medical male circumcision program: The Spear & Shield program
Source: PLOS Glob Public Health. 2024 Jan 31;4(1):e0002065. doi: 10.1371/journal.pgph.0002065 (PMC10830021; doi:10.1371/journal.pgph.0002065)
Supplement: Attachment — Submitted filename: RR SS2 Main Paper.docx [file pgph.0002065.s004.docx]

Thank you for the opportunity to revise this manuscript. Below, we provide a detailed response to the reviewers along with revisions to the manuscript. We hope these revisions are acceptable for publication in PLOS One Global Health.

Journal Requirements:

1. Please review your reference list to ensure that it is complete and correct. If you have cited papers that have been retracted, please include the rationale for doing so in the manuscript text, or remove these references and replace them with relevant current references. Any changes to the reference list should be mentioned in the rebuttal letter that accompanies your revised manuscript. If you need to cite a retracted article, indicate the article’s retracted status in the References list and also include a citation and full reference for the retraction notice.

**Response:** We reviewed the reference list. We do not have any retracted articles cited.

2. Please amend your detailed Financial Disclosure statement. This is published with the article. It must therefore be completed in full sentences and contain the exact wording you wish to be published.

**Response:** We added a financial disclosure statement.

a. State the initials, alongside each funding source, of each author to receive each grant.
b. If any authors received a salary from any of your funders, please state which authors and which funders.

**Response:** We added the initial of the authors who received the grant, if applicable. The PI of the P30 listed is not on the author list.

If you did not receive any funding for this study, please simply state: “The authors received no specific funding for this work.”

Additional Editor Comments (if provided):


[Note: HTML markup is below. Please do not edit.]

Reviewers' comments:

Reviewer's Responses to Questions

**Comments to the Author**

Reviewer #1: (No Response)

Reviewer #3: 1. Introduction needs updating.
Close to 30M VMMC had been performed by 2020, averting 615, 000 new HIV infections. With the 29.6M VMMC conducted by 2020, if we stop circumcising now, 4.9 M new HIV infections will be averted by 2030. (Uneven progress on the voluntary medical male circumcision. Programme across 15 eastern and southern African countries in the face of the COVID-19 pandemic. Geneva: UNAIDS & WHO; 2022). Recently, at AIDS Impact, # of VMMC was reported to be >30M and even more new infections averted? (contact [zembel@unaids.org](mailto:zembel@unaids.org) for latest figures).

**Response:** We have cited the citation provided and updated the introduction as suggested. Thank you.

Models have shown that VMMC remains a cost-effective, often cost-saving, prevention intervention in sub-Saharan Africa for at least the next 5 years (Cost-effectiveness of voluntary medical male circumcision for HIV prevention across sub-Saharan Africa: results from five independent models. Lancet Glob Health. 2023). Concerns were whether it was still effective given scale-up of HIV treatment (for prevention and treatment) programs).

**Response:** We have cited the citation provided and refererenced it in text as well as suggested. Thank you.

2. Ethical approval - include approvals numbers (e.g. Prior to study initiation, investigators obtained approval from the Research Ethics Committee in Zambia (University of Zambia, #XXX), from the provincial and district leadership, and from the affiliated US Institutional Review Board (University of Miami, #YYY).

**Response:** We have added the approval numbers, as requested.

3. Minor edits
Innovative VMMC programs such as S&S2 can improve the uptake VMMC, of one of the most...?

**Response:** We have corrected this.

VMMC program until completion of the study (no full stop).

**Response:** We have corrected this.

Reviewer #4: Summary
The manuscript describes the programmatic roll out of interventions derived from the Spear and Shield (S & S) clinical trial to four provinces in Zambia. S&S intervention was associated with increased likelihood to of circumcision uptake and higher rate of condom use. Its programmatic roll out was implemented from Nov 2015 to December 2020 to promote uptake of VMMC. Authors assessed the roll out using RE-AIM model to measure VMMC reach, uptake, and other attributes of program performance. They reported an increase in number of circumcisions at clinic level and concluded that the program successfully utilized the RE-AIM model to achieve study goals for implementation and dissemination.

General comments:
The manuscript raises important considerations for improving VMMC program update by matching demand creation with service availability. It however requires edits to improve clarity of the link Objectives, methods, results and conclusions. It would be helpful to start by crystalizing the objectives.

**Response:** We have clarified the objectives in the context of the RE-AIM model for clarity.


Specific Comments
Abstract:
The results do not clearly show how the RE-AIM model helped achieve the study goals for implementation and dissemination as stated in the conclusion. The authors should edit the abstract to show a clear link between objectives, RE-AIM Model, results and conclusion. The results of assessing the VMMC program roll out against each element of RE-AIM framework should be made clearer.

**Response:** We have mapped the RE-AIM model to the results and the abstract, as requested.

Statistical analysis
A found it difficult to align the different fragmentations of the analysis with the objectives and the RE AIM framework. I am not sure if it can be simplified for the readers.

**Response:** We have clarified the objectives in the context of the RE-AIM model in the abstract, introduction, results, and discussion, which we believe will help with understanding the presentation of the results.
