# Supplementary material for: Dissemination and implementation of an evidence-based voluntary medical male circumcision program: The Spear & Shield program
Source: PLOS Glob Public Health. 2024 Jan 31;4(1):e0002065. doi: 10.1371/journal.pgph.0002065 (PMC10830021; doi:10.1371/journal.pgph.0002065)
Supplement: Attachment — Submitted filename: Response to Reviewers 11.27.docx [file pgph.0002065.s005.docx]

Thank you for the opportunity to revise this manuscript. Below, we provide a detailed response to the reviewers along with revisions to the manuscript. We hope these revisions are acceptable for publication in PLOS One Global Health.

Journal Requirements:

1. Please review your reference list to ensure that it is complete and correct. If you have cited papers that have been retracted, please include the rationale for doing so in the manuscript text, or remove these references and replace them with relevant current references. Any changes to the reference list should be mentioned in the rebuttal letter that accompanies your revised manuscript. If you need to cite a retracted article, indicate the article’s retracted status in the References list and also include a citation and full reference for the retraction notice.

**Response:** We reviewed the reference list. We do not have any retracted articles cited.

2. Please include information in the Methods section on type of consent obtained, and whether the IRBs approved the consent procedures." 2) “Please include a complete copy of PLOS’ questionnaire on inclusivity in global research in your revised manuscript. Our policy for research in this area aims to improve transparency in the reporting of research performed outside of researchers’ own country or community. The policy applies to researchers who have travelled to a different country to conduct research, research with Indigenous populations or their lands, and research on cultural artefacts. The questionnaire can also be requested at the journal’s discretion for any other submissions, even if these conditions are not met.  Please find more information on the policy and a link to download a blank copy of the questionnaire here: [https://journals.plos.org/plosone/s/best-practices-in-research-reporting](https://urldefense.com/v3/__https:/journals.plos.org/plosone/s/best-practices-in-research-reporting__;!!KVu0SnhVq1hAFvslES2Y!PKlXo9gwQ9QXFHuefI9Hzbw0S6RcwLxIpVqS5jgyXvP9aECaCmh1XKwpuJ9masbYBtykyv7xcPchP4ZSvAUHZQ$). Please upload a completed version of your questionnaire as Supporting Information when you resubmit your manuscript.

**Response:** We have uploaded the completed questionnaire as Supporting Information.

Additional Editor Comments (if provided):


[Note: HTML markup is below. Please do not edit.]

Reviewers' comments:

Reviewer's Responses to Questions

**Comments to the Author**

1. If the authors have adequately addressed your comments raised in a previous round of review and you feel that this manuscript is now acceptable for publication, you may indicate that here to bypass the “Comments to the Author” section, enter your conflict of interest statement in the “Confidential to Editor” section, and submit your "Accept" recommendation.

Reviewer #3: All comments have been addressed

Reviewer #4: All comments have been addressed

2. Does this manuscript meet PLOS Global Public Health’s [publication criteria](https://urldefense.com/v3/__https:/journals.plos.org/globalpublichealth/s/criteria-for-publication__;!!KVu0SnhVq1hAFvslES2Y!PKlXo9gwQ9QXFHuefI9Hzbw0S6RcwLxIpVqS5jgyXvP9aECaCmh1XKwpuJ9masbYBtykyv7xcPchP4ZJxSeLuw$)? Is the manuscript technically sound, and do the data support the conclusions? The manuscript must describe methodologically and ethically rigorous research with conclusions that are appropriately drawn based on the data presented.

Reviewer #3: Yes

Reviewer #4: Yes

3. Has the statistical analysis been performed appropriately and rigorously?

Reviewer #3: Yes

Reviewer #4: Yes

4. Have the authors made all data underlying the findings in their manuscript fully available (please refer to the Data Availability Statement at the start of the manuscript PDF file)?

The [PLOS Data policy](https://urldefense.com/v3/__https:/journals.plos.org/globalpublichealth/s/data-availability__;!!KVu0SnhVq1hAFvslES2Y!PKlXo9gwQ9QXFHuefI9Hzbw0S6RcwLxIpVqS5jgyXvP9aECaCmh1XKwpuJ9masbYBtykyv7xcPchP4ZV4bkZOQ$) requires authors to make all data underlying the findings described in their manuscript fully available without restriction, with rare exception. The data should be provided as part of the manuscript or its supporting information, or deposited to a public repository. For example, in addition to summary statistics, the data points behind means, medians and variance measures should be available. If there are restrictions on publicly sharing data—e.g. participant privacy or use of data from a third party—those must be specified.

Reviewer #3: Yes

Reviewer #4: Yes

5. Is the manuscript presented in an intelligible fashion and written in standard English?

PLOS Global Public Health does not copyedit accepted manuscripts, so the language in submitted articles must be clear, correct, and unambiguous. Any typographical or grammatical errors should be corrected at revision, so please note any specific errors here.

Reviewer #3: Yes

Reviewer #4: Yes

6. Review Comments to the Author

Please use the space provided to explain your answers to the questions above. You may also include additional comments for the author, including concerns about dual publication, research ethics, or publication ethics. (Please upload your review as an attachment if it exceeds 20,000 characters)

Reviewer #3: All comments have been addressed.

Reviewer #4: No further comments

7. PLOS authors have the option to publish the peer review history of their article ([what does this mean?](https://urldefense.com/v3/__https:/journals.plos.org/globalpublichealth/s/editorial-and-peer-review-process*loc-peer-review-history__;Iw!!KVu0SnhVq1hAFvslES2Y!PKlXo9gwQ9QXFHuefI9Hzbw0S6RcwLxIpVqS5jgyXvP9aECaCmh1XKwpuJ9masbYBtykyv7xcPchP4bAFQXWSw$)). If published, this will include your full peer review and any attached files.


**Do you want your identity to be public for this peer review?** If you choose “no”, your identity will remain anonymous but your review may still be made public.


For information about this choice, including consent withdrawal, please see our [Privacy Policy](https://urldefense.com/v3/__https:/www.plos.org/privacy-policy__;!!KVu0SnhVq1hAFvslES2Y!PKlXo9gwQ9QXFHuefI9Hzbw0S6RcwLxIpVqS5jgyXvP9aECaCmh1XKwpuJ9masbYBtykyv7xcPchP4Y-9etTJg$).

Reviewer #3: No

Reviewer #4: No
